# Electrophysiological measurement of ion channels on plasma/organelle membranes using an on-chip lipid bilayer system

**DOI:** 10.1038/s41598-018-35316-4

**Published:** 2018-11-30

**Authors:** Koki Kamiya, Toshihisa Osaki, Kenji Nakao, Ryuji Kawano, Satoshi Fujii, Nobuo Misawa, Masatoshi Hayakawa, Shoji Takeuchi

**Affiliations:** 1 Artificial Cell Membrane Systems Group, Kanagawa Institute of Industrial Science and Technology, 3-2-1 Sakado Takatsu-ku, Kawasaki, Kanagawa 213-0012 Japan; 2 Institute of Industrial Science, The University of Tokyo, 4-6-1 Komaba, Meguro-ku, Tokyo 153-8505 Japan; 3 Biomolecular Research Laboratories, Pharmaceutical Research Division, Takeda Pharmaceutical Company Ltd., 2-26-1 Muraokahigashi, Fujisawa, Kanagawa 251-8555 Japan; 4 Research and Development Department, Kanagawa Institute of Industrial Science and Technology, 3-2-1 Sakado, Takatsu-ku, Kanagawa 213-0012 Japan

## Abstract

Ion channels are located in plasma membranes as well as on mitochondrial, lysosomal, and endoplasmic reticulum membranes. They play a critical role in physiology and drug targeting. It is particularly challenging to measure the current mediated by ion channels in the lysosomal and the endoplasmic reticulum membranes using the conventional patch clamp method. In this study, we show that our proposed device is applicable for an electrophysiological measurement of various types of ion channel in plasma and organelle membranes. We designed an on-chip device that can form multiple electrical contacts with a measurement system when placed on a mount system. Using crude cell membranes containing ion channels extracted from cultured cells without detergents, we detected open/close signals of the hERG, TRPV1, and NMDA channels on plasma membranes, those of the TRPML1 channels on lysosomal membranes, and open/close signals of the RyR channels on SR membranes. This method will provide a highly versatile drug screening system for ion channels expressed by various cell membranes, including plasma, SR, mitochondrial, Golgi, and lysosomal membranes.

## Introduction

Ion channels in cell membranes form pores that facilitate the transport of ions (sodium, potassium, calcium, and chloride) across membranes^1^. They have attracted a considerable amount of research interest in the fields of physiology^2^, pharmacology^3^, and pathophysiology^4^. The functions of a single ion channel have been investigated based on current using an electrophysiological technique known as the patch clamp method^5–7^. This technique involves manual operation to patch a glass microcapillary to the surface of the cell membrane to form a high-resistance seal in the giga-ohm range. This procedure requires skill and patience on part of the operators. Moreover, it is challenging to use the patch clamp technique to study ion channels located in the membranes of subcellular organelles; including mitochondria, Golgi, and lysosome; because the capillary cannot access organelle membranes. Artificial planar bilayer lipid membrane (BLM) systems have recently been developed to study the ion channel current in a more engineering-oriented approach^8,9^. In these systems, the target ion channels are purified from living cells, reconstituted in the BLM, and ion current is measured by a patch clamp amplifier. In past research, we have developed a parallel ion channel recording device with 16 separate channels (16-ch) of BLM wells^10^, where the bilayers in the wells are formed based on the droplet contact method^11^. The device allows for the screening of ion channels, where inhibitors (drugs) can be arbitrarily applied to the intra-cellular- and/or extra-cellular side of the ion channels at a particular time^10^.

**Figure 1:**
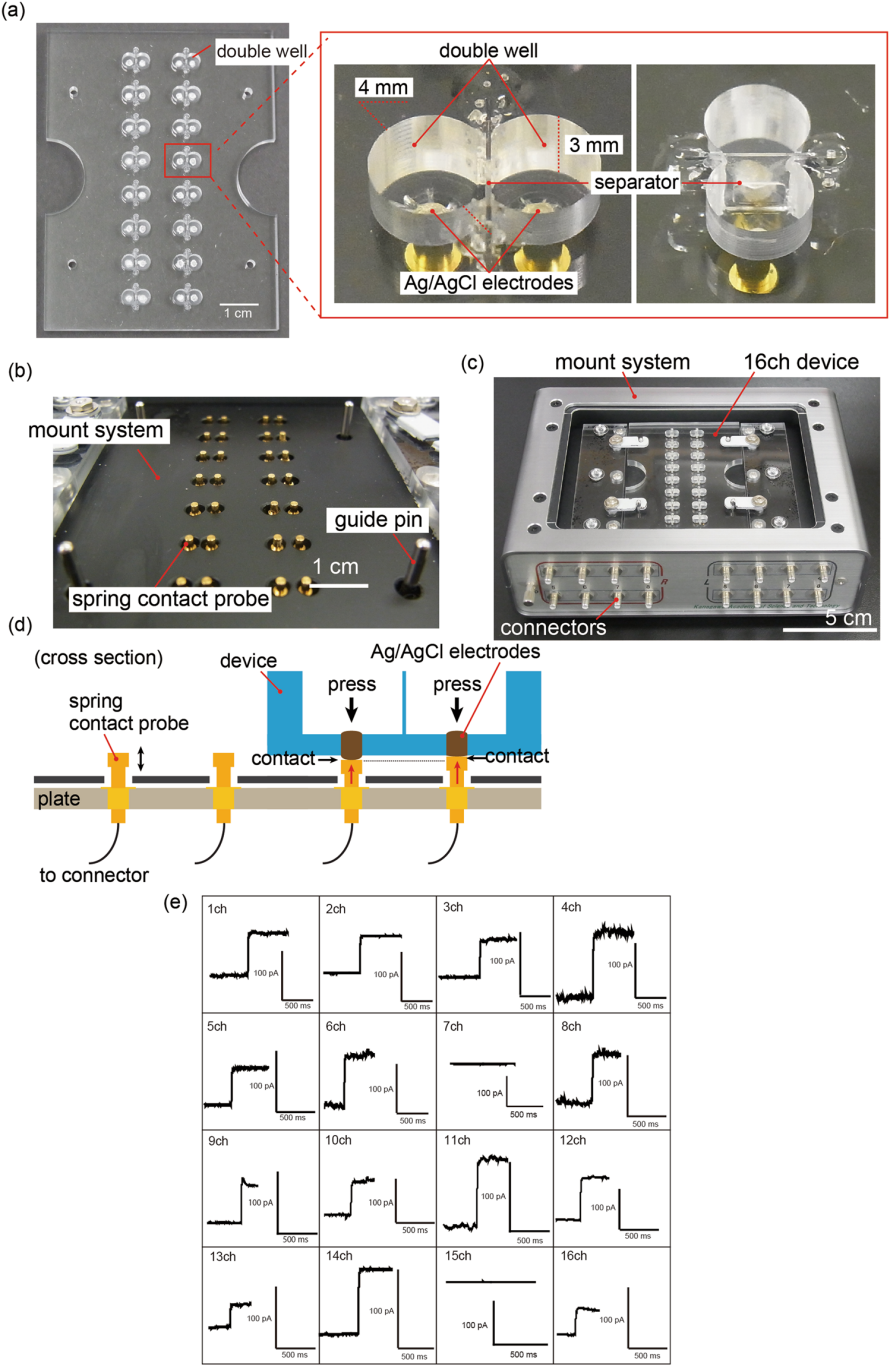
(**a**) 16-ch BLM chip and magnified images of the double well. The double well was micromachined on an acrylic basement and the separator was inserted in its middle. Ag/AgCl electrodes were placed at the bottom of the well. (**b**) Spring contact probes integrated into the mount system. (**c**) Photograph of 16-ch BLM chip equipped on the mount system. (**d**) Probes were pressed to each electrode from the bottom, and provided reliable electrical connection between the electrodes and the amplifier due to the spring. (**e**) Current traces of α-hemolysin nanopore using our system. Applied potential: +100 mV.

In this paper, we modify our previously developed device to improve its reliability and make it easier to handle for electrical measurements, and apply it to the electrophysiological measurement of various types of ion channels on plasma and organelle membranes (the human Ether-a-go-go-Related Gene (hERG)^12,13^, Transient Receptor Potential Vanilloid 1 (TRPV1)^14^, N-methyl-D-aspartate (NMDA)^15^, Ryanodine Receptor (RyR)^16^, and Transient Receptor Potential Mucolipin 1 (TRPML1)^17^). The on-chip BLM system consists of a simplified 16-ch BLM chip and a mount system equipped with 16 electric contact probes, such that parallel current can be recorded simply by placing the chip on the mount system, as showing in Fig. 1(a–d). To show the applicability of the device to a variety of ion channels, we focus on crude cell membrane fractions prepared from living cells that express the target ion channel. These membranes were prepared by sonication and centrifugation in the absence of detergents, which suppressed the detergent-mediated denaturation of the ion channels. Current through the ion channels was measured by reconstitution of ion channels using fusion of the crude cell membrane fraction with BLM. Using our device, we recorded the ion current signals of voltage-gated ion channels, hERG, and ligand-gated ion channels, TRPV1 and NMDA, in plasma membranes. We also recorded the ion current signals of TRPML1 and RyR, which are normally expressed on lysosome membranes and SR membranes, respectively.

## Results

### Verification of the on-chip BLM system

To evaluate the usability and reliability of the on-chip system developed with 16-ch BLM wells (Fig. 1(a–d)), we recorded the ion current of a nanopore-forming protein (α-hemolysin). As it is a water-soluble protein and forms a nanopore on the BLM, α-hemolysin was suitable for the verification of the system. As described in the “Methods” section, the 16-ch BLM chip was set on the mount system and the multichannel patch clamp amplifier was used to record the current. The 16-ch BLM chip was improved in terms of the connection between the 16-ch BLM chip, which contains Ag/AgCl electrodes at the bottom of the double well, and the contact probes, which were connected to the 16-ch amplifiers (Fig. 1b). The previous 16-ch BLM chip was wired by using Ag vapor deposition on the back side of the 16-ch BLM chip and the Ag wires were connected to the 16-ch amplifiers using special joining terminals^10^ (Fig. S1). Therefore, we consider the extent to which the wells of the improved BLM chips are disconnected to be less than that of the previously reported BLM chips. The BLM was formed at each double well using the droplet contact method. Here, 1 M of KCl with 10 mM of phosphate buffer (pH 7) containing α-hemolysin (200 nM) was applied to both droplets A and B. To rapidly form the BLM, we stroked the contact area of the droplets using a hydrophobic stick before signal measurements of α-hemolysin. Through time-course monitoring, we observed specific stepwise signals of α-hemolysin nanopores at 14 wells of the 16 channels (Fig. 1(e)). Current amplitudes of approximately 50 and 100 pA were obtained at +100 mV of applied voltage^18^. The results verified that the on-chip BLM system can be used to measure the current flowing through the ion channels.

We also improved the previously reported 16-ch BLM chip to increase the detection speed of the biological nanopores or ion channels in the BLM. The number of pores on the parylene film in the 16-ch chip was changed from 5 to 11 to increase the area available for the formation of the planar lipid bilayer (Fig. S2). The time at which the initial αHL current signal appeared was examined using two different 16-ch chips with either 5 pores or 11 pores without the BLM reform manner. The initial αHL current signal appeared sooner when the 16-ch device with 11 pores was used, compared to the device with 5 pores (Fig. S3). The results suggest that the difference in the number of pores (lipid bilayers) affects the probability of nanopore detection.

### Current measurement of plasma membrane ion channels

The current signals of the ion channels present in the plasma membrane *in vivo* were recorded using the proposed on-chip BLM system. The target ion channels were obtained as crude membrane fractions as described in the “Methods” section (see Fig. S4(a)). The BLM was formed on the 16-ch chip connected to a patch clamp amplifier. The crude membrane fractions were initially added to the droplet (see Table 1). By the fusion of the crude membrane fractions with the BLM, the target ion channels were incorporated into the BLM spontaneously (Fig. S4(b)). Although the crude membrane fractions were mainly composed of the overexpressed ion channels, the fractions also contained small amounts of the various endogenic ion channels from the host cells. For example, we used western blotting analysis to confirm the incorporation of hERG proteins in the crude membrane fractions (Fig. S5). As the BLM system observes the signals from a single ion channel, the contaminating proteins, even if in small amounts, could have disturbed the signal recording of the target ion channels. For this reason, we determined whether the signals of the specific target ion channels could be detected using the crude membrane fractions.

**Table 1:** Buffer solution and inhibitor conditions for respective ion channel recordings.

Ion channel	hERG	TRPV1	NMDA	TRPML1	RyR
Buffer composition of droplet A	10 mM HEPES 120 mM KCl (pH 7.2)	10 mM HEPES 140 mM NaCl 5 mM KCl 2 mM CaCl_2_ 2 mM MgCl_2_ 10 mM glucose 1 μM capsaicin (pH 7.4)	10 mM HEPES 150 mM NaCl 2.5 mM KCl 10 mM glutamate 10 mM glycine (pH 8.0)	10 mM HEPES 150 mM KCl (pH 7.4)	20 mM HEPES 250 mM KCl 200 μM CaCl_2_ (pH 7.4)
Buffer composition of droplet B	10 mM HEPES 120 mM KCl (pH 7.2)	10 mM HEPES 140 mM KCl 5 mM EGTA (pH 7.4)	10 mM HEPES 135 mM KCl 1 mM CaCl_2_ (pH 7.4)	10 mM HEPES 150 mM KCl (pH 7.4)	20 mM HEPES 250 mM KCl 3 mM ATP 10 μM CaCl_2_ 4 mM caffeine (pH 7.4)
Addition droplet of crude membrane fraction	Both Droplet	Droplet B	Droplet A	Droplet A	Droplet B
Inhibitor concentration and droplet topology	100 nM astemizole in Droplet B 200 μM E-4031 in both droplets	100 μM capsazepine in Droplet A	100 μM MK-801 in Droplet A 5 mM MgCl_2_ in both droplets	1 mM verapamil in Droplet A	500 nM ruthenium red in both droplets

**Figure 2:**
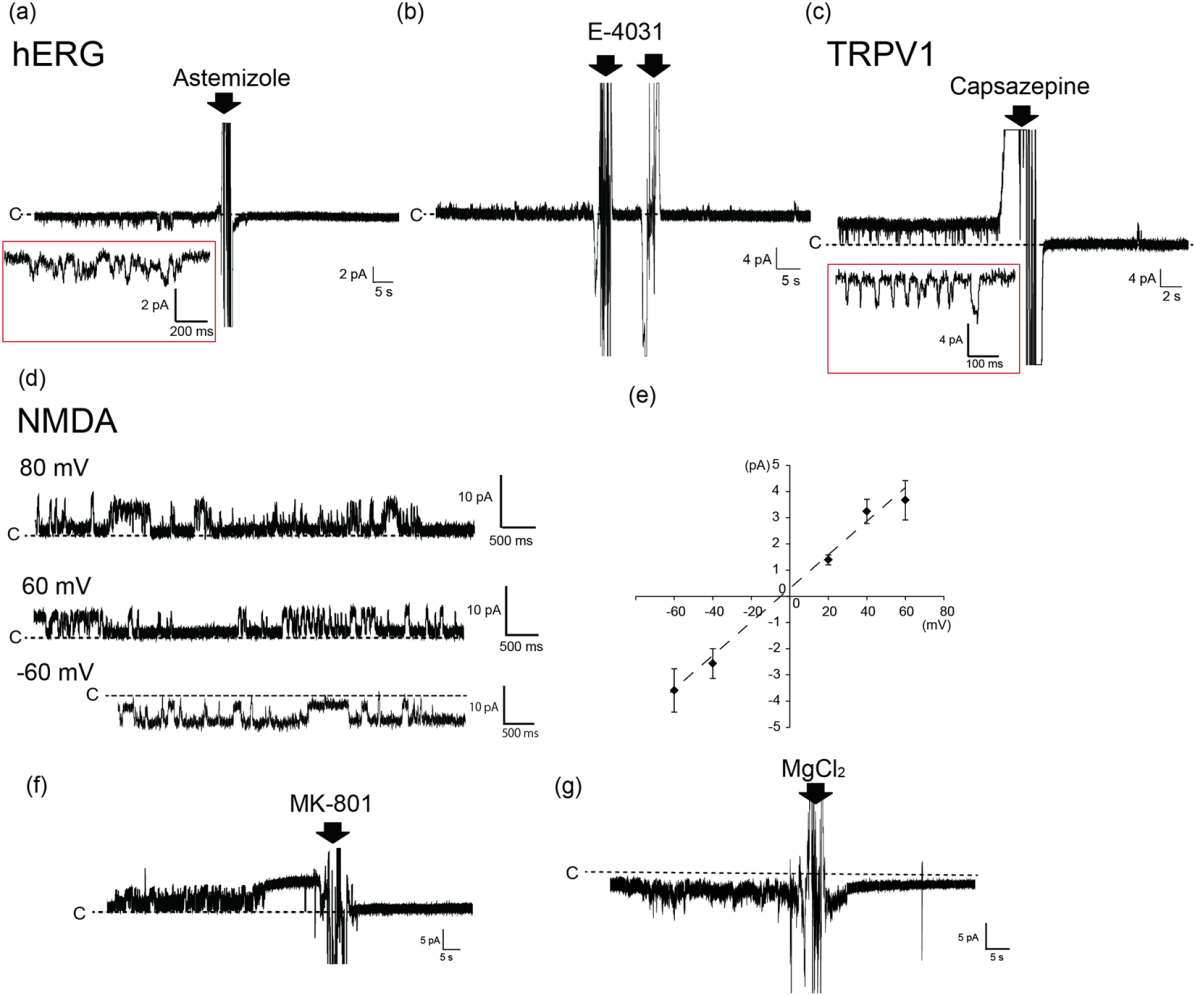
(**a**,**b**) Current traces of the hERG channel with the addition of hERG inhibitors astemizole and E-4031. The final concentrations of astemizole and E-4031 were 100 nM and 200 μM, respectively. The red square represents an expansion of the hERG signal. The arrows represent the addition of inhibitors. Applied potential: −80 mV (**a**) and + 60 mV (**b**). (**c**) Current trace of TRPV1 channel with the addition of the TRPV1 inhibitor, capsazepine. The red square represents an expansion of the TRPV1 signal. The arrow represents the addition of capsazepine. The final concentration of capsazepine was 100 μM. Applied potential: + 60 mV. (**d**) Current trace of the NMDA channel. Applied potential: -60 mV, + 60 mV, and + 80 mV. (**e**) I-V curve of the NMDA channel. Error bars: s.d. (**f**) Inhibition of the NMDA channel signal by the NMDA inhibitor, MK-801. The arrow represents the addition of MK-801. The final concentration of MK-801 was 100 μM. Applied potential: +80 mV. (**g**) Inhibition of the NMDA channel signal by magnesium ion. The arrow represents the addition of MgCl_2_. The final concentration of MgCl_2_ was 5 mM. Applied potential: −80 mV.

We first conducted observations of the open/close signals of a potassium channel encoded by the human Ether-a-go-go Related Gene (hERG), followed by that of the blocking of the signals in the presence of specific inhibitors. The hERG channel consisted of a homogeneous tetramer. We obtained a current amplitude of approximately −0.8 ± 0.2 (mean ± s.d.) pA at -80 mV with 120 mM of KCl (Fig. 2(a,b)). This current amplitude was similar to the result that was previously reported for hERG ion channels in a BLM system^19^. After consistently observing this current signal, we introduced the specific hERG inhibitors astemizole or E-4031. An antihistamine, astemizole causes the prolongation of the QT interval because of the blocking of the function of the hERG channel^20^. E-4031 is a methanesulfonanilide Class III antiarrhythmic drug that inhibits the function of hERG-type potassium channels, and shows a delay in the QT interval. Following the addition of astemizole with a final concentration of 100 nM to droplet B (the ground well), the open current signals disappeared (Fig. 2(a)). This inhibition further supported the claim that the observed signal came from the hERG channel. An advantage of the proposed system is that such reagents as inhibitors can be added to respective wells at desired timings because every double well is isolated on the chip. For example, when three hERG-like signals appeared at different time points on a single 16-ch chip, we were able to conduct three inhibition assays one by one (Fig. S6). This type of assay was not available in previous systems^21^. Similar results were observed after the addition of E-4031 with a final concentration of 200 μM of both droplets A and B (Fig. 2(b))^19^. Using crude membrane fractions without hERG expression, we detected a few open/close current signals. Even in the few cases that we did, the signals were not inhibited by the addition of the hERG inhibitor (astemizole) (Fig. S7), suggesting that the signals had been derived from endogenic ion channels. Using the patch-clamp method of the artificial lipid bilayer, Hirano group and Schmidt group separately reported that the single current signals of the hERG channels were successfully detected using the crude membrane fraction because this fraction, which was formed from the hERG channels-overexpressed cells, contains a large amount of these channels^13,19^. Taken together, these results suggest that the observed open/close current signals were hERG channels using crude membrane fractions. The number of ion channels detected in the 16-ch BLM chip is approximately 1–3 wells (seven independent experiments) (Fig. S6) because the incorporation of the ion channels into the BLM rely on a conventional vesicle fusion method^10^. Therefore, in the next step, the efficiency of the ion channel current detection can be increased by improving the frequency of the vesicle fusion, by changing the lipid components of the planar lipid bilayer^22^, or by adding the membrane fusion-induced peptide such as fusion-induced peptide^23^ or complementary DNA^24^. Another idea would be to use BLM chips with more than 16 channels to increase the number of ion channels that could be used for current detection.

Following the above, we applied the transient receptor potential vanilloid 1 (TRPV1) channels to the BLM system using crude membrane fractions. TRPV1 is activated by such ligands as capsaicin, and by heat and low pH. In the presence of 1-μM capsaicin in droplet A (the recording well), we observed current signals typical of TRPV1 channels with dominant open states. It has been reported in past research that open signals become the major state in the presence of approximately 1 μM of capsaicin^25^. We observed a current amplitude of 2.9 ± 0.4 (mean ± s.d.) pA at + 60 mV with 140 mM of KCl (droplet B) and 140 mM of NaCl (droplet A) (Fig. 2(c); see also Table 1). This current amplitude was similar to the result observed previously^26^. Subsequently, a final concentration of 100 μM of capsazepine (a TRPV1 inhibitor) was added to droplet A (the recording well)^27^ because capsazepine is known to work in the extracellular domain of the TRPV1^28^. We observed the disappearance of open ion current. Therefore, the inhibition of the TRPV1 that we observed was oriented toward the intracellular side of droplet B in the BLM. These results suggest that TRPV1 on the crude membrane fractions exposes its intracellular domain to the outside.

Moreover, we tested the ligand-gated NMDA (N-methyl-D-aspartate) receptor, which consisted of a heterogeneous tetramer channel, with our system. In the presence of 10 mM glutamate, 10 mM glycine, and the crude cell membrane fraction containing NMDA in droplet A (the recording well), we obtained current amplitudes of 3.7 ± 0.7 (mean ± s.d.) pA and −3.6 ± 0.8 (mean ± s.d.) pA at + 60 mV and −60 mV, respectively (Fig. 2(d,e)). These current amplitudes are consistent with the previous data reported for the NMDA receptor in the liposome or the cell^29,30^. The current signals were inhibited by the NMDA receptor inhibitor, 100 μM MK-801, added to droplet A (the recording well)^31^. MK-801 is known to work in the extracellular domain of NMDA channels (Fig. 2(f))^32^. The result indicates that there may be an extracellular domain-exposed orientation of NMDA channels in the crude membrane fractions because both the crude membrane fractions with NMDA and the inhibitor were added to the same droplet A. Magnesium ion also works as the inhibitor of NMDA proteins. The magnesium ion is binding to the pore of NMDA channels. The current signals of the NMDA channels were inhibited by adding 5 mM MgCl_2_ at final concentration to each droplet (Fig. 2(g)). The NMDA current signals, which were obtained using 16-ch chips, were blocked using two kinds of inhibitors.

### Current measurement of ion channels present in organelle membranes

We further verified the applicability of the proposed BLM system in combination with the fusion method of crude membrane fractions that specifically contains ion channels present in organelle membranes, such as mitochondria, Golgi, and the endoplasmic reticulum. Few studies have reported the electrophysiology of ion channels in organelle membranes owing to technical difficulties^33^. These membranes are challenging to access with a glass capillary in patch clamp methods because of the size of the organelle (ca. 100–300 nm). BLM systems have been applied to study ion channels in the organelle membrane—for example, mitochondrial ion channels (voltage-dependent anion selective channel: VDAC) purified by detergents^34,35^. Ion channels in the lysosomal and SR membranes have also been studied with the conventional patch clamp method by conjugating them with a plasma membrane transfer signal, which facilitated the expression of the channels on the plasma membranes^36^. We measured the current in the ion channels containing a lysosome and an SR membrane using the crude membrane fraction fusion method.

**Figure 3:**
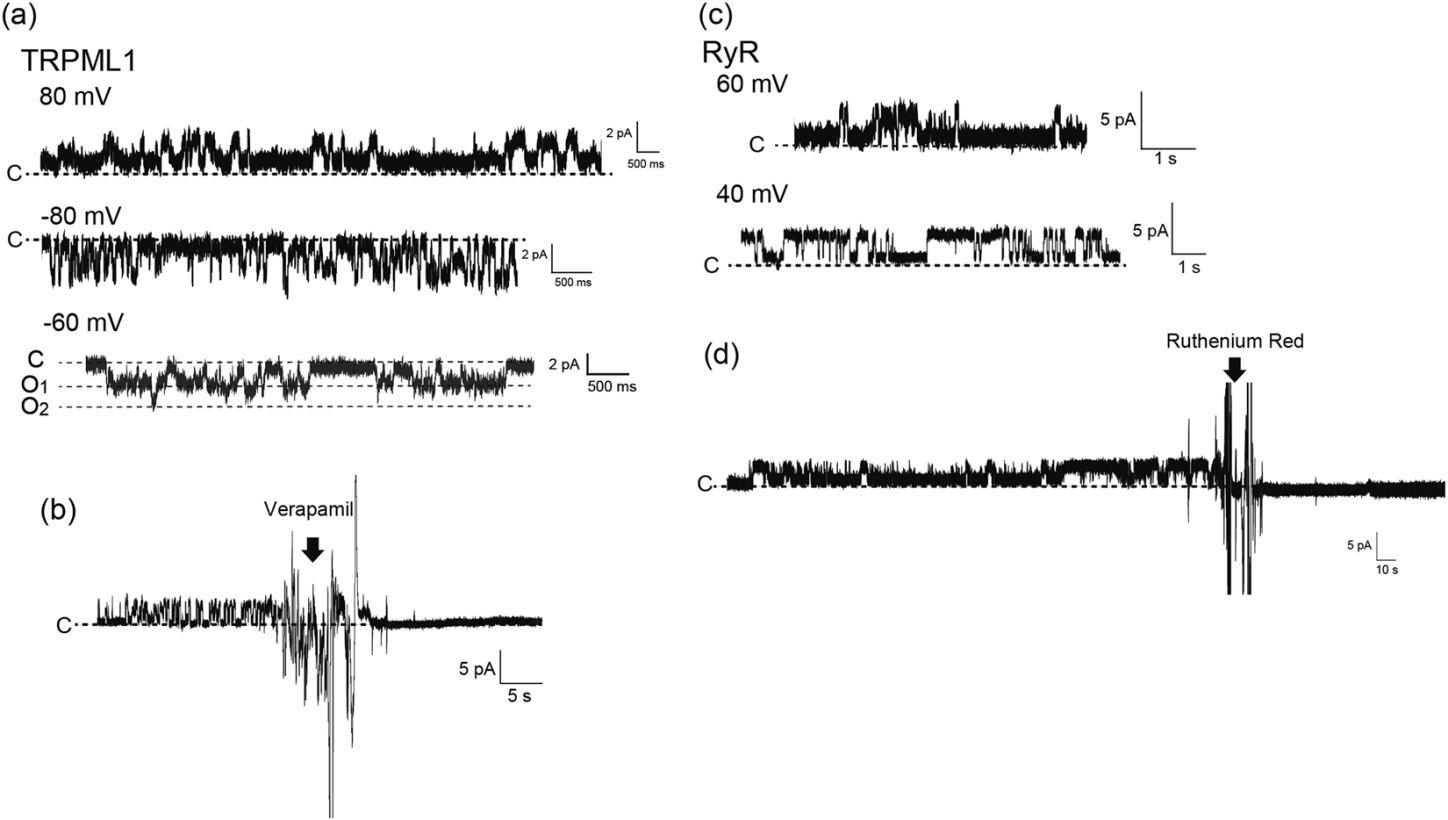
(**a**) Current traces of the TRPML1 channel. Applied potential: +80 mV, −60 mV, and −80 mV. (**b**) Inhibition of TRPML1 channel signal by the TRPML1 inhibitor, verapamil. The arrow represents the addition of verapamil. The final concentration of verapamil was 1 mM. Applied potential: +80 mV. (**c**) Current trace of the RyR channel. Applied potential: +40 mV and +80 mV. (**d**) Inhibition of the RyR channel’s signal by the RyR inhibitor, ruthenium red. The arrow represents the addition of ruthenium red. The final concentration of ruthenium red was 500 nM. Applied potential: +40 mV.

First, we conducted the signal recordings of transient receptor potential mucolipin1 (TRPML1) channels expressed in lysosomal membranes. The crude cell membrane fractions containing the plasma and lysosomal membranes were isolated from the overexpressed TRPML1 cells. The crude cell membrane fraction containing TRPML1 was added to droplet A (the recording well). We obtained current amplitudes of 1.2 ± 0.2 (mean ± s.d.) pA and −1.7 ± 0.3 (mean ± s.d.) pA at +80 mV and −80 mV, respectively, in the presence of 150 mM of KCl (both droplets) (Fig. 3(a)). Current amplitudes of approximately 1.6 pA at +80 mV with 150 mM of KCl have been previously reported on a BLM system^37^. The open/close signals of the TRPML1 channels were inhibited by 1 mM of verapamil, which was added only to droplet A (the recording well)^17^. This result suggests that verapamil worked in the extracellular domain of the TRPML1 channels (Fig. 3(b)). Therefore, there might have been an extracellular domain-exposed orientation of TRPML1 channels into the crude membrane fractions.

We further focused on the ryanodine receptor (RyR) in SR membranes isolated from the canine heart. RyR on the SR membrane formed a calcium release channel in the SR membranes. Calcium release by RyR channels was activated by the addition of ATP or caffeine to the cytoplasm side of the RyR channels^38,39^. To define the orientation of the RyR channels in the BLM, we applied different components of a solution in droplets A and B as shown in Table 1. ATP and caffeine were added to droplet B while a higher concentration of CaCl_2_ was added to droplet A. SR membranes containing the RyR channels were added to droplet B. In this system, we oriented the cytoplasmic side of RyR channels in droplet B. We obtained the open/close signals with a current amplitude of 1.8 ± 0.2 (mean ± s.d.) pA at +40 mV (Fig. 3(c)). As an inhibition test of the calcium release of the RyR channels, 500 nM of ruthenium red was added to both droplets, and we observed the blocking of the RyR channel’s open/close signals (Fig. 3(d))^40^. These results suggest that the calcium release activities of RyR channels can be observed in our BLM system.

## Discussion

Combined with the crude cell membrane fractions containing the target ion channels, as a proof of concept, the capability of the proposed on-chip BLM system was verified in terms of signal detection for various types of ion channels obtained from the plasma and organellar membranes. As shown in the results, our approach allowed for the identification of the target ion channels, and showed potential for screening candidate drugs in ion channels present in the plasma, SR, mitochondrial, Golgi, and lysosomal membranes^33,41,42^.

The proposed on-chip BLM system provides the following advantages. First, it provides adequate data throughput to record the current flowing through ion channels. The conventional patch clamp method suffers from problems relating to throughput as the operator needs to patch cultured cells one by one. By contrast, our BLM chip, which currently accommodates 16 independent current recordings, is scalable to realize high-throughput drug screening of ion channels on the plasma and organelle membranes. Although a commercially available cell-based patch-clamp platform such as SynchroPatch allows hundreds of experiments to be conducted in parallel in a highly automated fashion, current measurements of the ion channels on the organelle membranes using the automated cell-based patch-clamp platform are difficult. Second, the independent, parallel recording also facilitates assays of ion channels with low probabilities of appearance over signal recordings. For example, although just three hERG-like signals appeared during a two-hour assay, the inhibition assay was successfully conducted separately for all three points (Fig. S6). Third, the double-well chip helps determine the topological directions of the actions of the inhibitor on the target ion channels incorporated into the BLM, as both sides of the aqueous solutions (droplets) are independently accessible^10^. Each droplet corresponds to the inside and outside of the membrane environment, respectively. We presented examples of possible topological directions of TRPV1, NMDA, and TRPML1 by using specific extra- and intra-cellular inhibitors. Fourth, the double-well chip can form an asymmetric lipid bilayer similar to that of the eukaryotic cell membranes^43,44^. Our device can be used to elucidate new functions/characteristics of ion channels under lipid compositions mimicking cell membranes. In summary, our BLM system is more flexible than those of other multiple-BLM array devices; in commercially available artificial bilayer-based patch-clamp systems with multiple parallel recordings such as Orbit 16, it is difficult to add drugs to both sides (the inside and outside of the membrane environment) and to conduct some inhibition assays one by one.

The use of crude cell membrane fractions is another feature of this study. Our approach is applicable to various types of ion channels. The conductance of the measured ion channels was in good agreement with previously reported ones. The currents through the ion channel were inhibited by the respective inhibitors in accordance with previously reported concentrations. Thus, the ion channels incorporated into the BLM retained their native functions. The versatility might have obtained because the crude membrane fractions were prepared from the ion channels-expressing cells using sonication and centrifugation without detergents, which avoided their detergent-mediated denaturation. Moreover, heterogeneous domain-formed ion channels, such as NMDA, and intracellular (organelle) membrane-expressed ion channels, which are difficult to purify, can be easily prepared using crude membrane fractions. Moreover, this approach has an advantage in terms of sample amount. A volume of 0.2 mL of crude membrane fraction with a total protein concentration of approximately 0.1 mg/mL was obtained from approximately 6 × 10^7^ cells. Because the single double well consumed 20 µL with approximately 1 × 10^−4^ mg/mL protein concentration in an assay, the crude membrane fraction sample would available for 10 thousands double wells. The fractions can be stored at −80 °C for over six months. To prepare the proteoliposomes for artificial patch clamp measurement using the BLM, the target ion channels were purified from the large amount of target ion channels-expressed cells (0.5–1 L). Therefore, compared to an ion channel current measurement system that uses proteoliposomes, our system using the crude cell membrane fractions reduces the amount of sample required for analysis and maintains the ion channel functions. Moreover, the current measurements of ion channels using the crude cell membrane fractions obtain the averaging data of cell-to-cell variability (the average of the data set), compared to the single cell-based patch-clamp method. Therefore, if our system can achieve a high probability of ion channel detections, our system would be useful for analyzing the drug screening of the ion channels.

## Materials and Methods

### Materials

1,2-Dioleoyl-*sn*-glycero-3-phosphocholine (DOPC), and 1,2-dioleoyl-*sn*-glycero-3-phosphoethanolamine (DOPE) were purchased from Avanti Polar Lipids (Alabama, USA). E-4031, capsazepine, and MK-801 were purchased from TOCRIS Bioscience (Bristol UK). Na_3_PO_4_, NaCl, KCl, CaCl_2_, MgCl_2_, glucose, sucrose, glycerol, capsaicin, ruthenium red, glutamate, glycine, caffeine, and ATP were purchased from Wako Pure Chemical Industries (Osaka, Japan). HEPES was purchased from Dojindo Molecular Technologies (Kumamoto, Japan), Verapamil was purchased from Sigma-Aldrich (St. Louis USA), and astemizole was purchased from Toronto Research Chemicals (North York, Canada).

### 16-ch BLM chip

A 16-ch BLM chip was assembled with three parts (Fig. 1(a)). An acrylic basement consisted of 16 pairs of double wells with a diameter of 4 mm and a depth of 3 mm, micromachined using a minimiller MM-100 (Modia Systems, Saitama, Japan). As electrodes, silver wires (1 mm in diameter) were embedded at through holes at the bottom of the wells. Ag/AgCl paste (BAS Inc., Tokyo, Japan) was deposited on top of the wire and desiccated at room temperature overnight. A perforated separator was inserted and glued between the double wells to restrict the area of the lipid bilayer that had formed. The separator was composed of a thin poly(chloro-*p*-xylylene) (parylene) film (5 μm thickness), sandwiched by a pair of an acrylic frame (75 μm thick) (Fig. S8). The microapertures were fabricated on a parylene film by using standard photolithography^10^. The BLM was suspended at microapertures on the film by the procedure described in the “Methods” section below.

### Mount system to connect 16-ch BLM chip

A reliable electrical connection between the electrodes on the BLM chip and the patch clamp amplifier is a critical factor in precise recordings of the ion channel. In this study, we develop an in-house mount system that enables such a connection when the chip is placed on it (Fig. 1(b,c)). The mount system is composed of spring contact probes commonly used to test circuit boards. The contact probes are aligned on a plastic plate such that the position of each probe head corresponds to the position of each electrode (Fig. 1(d)). The spring guarantees contact with every electrode on the chip by absorbing gaps formed by fabrication error and/or deformation of the plastic chip. The flat head of the probe is 1 mm in diameter and the travel distance (i.e., margin for the gap) is 2.5 mm. The back of the probe is wired to the connector, which is in turn connected to the amplifier. The aluminum chassis of the mount system was grounded for Faraday shield.

### Crude cell membrane fractions containing target ion channels

Each of hERG, NMDA, TRPV1 and TRPML1 plasmid was transfected into FreeStyle 293 cells using 293fectin transfection reagent (Thermo Fisher Scientific, Massachusetts, USA) following manufacturer’s protocol. Two days after transfection, the cells were collected and resuspended in a resuspension buffer (50 mM Na_3_PO_4_, 300 mM NaCl, 10 mM KCl, 8% (w/v) sucrose, 10% (v/v) glycerol, and the complete protease inhibitor (pH 7.0)). The solution was sonicated on ice and centrifuged at 10,000 × g for 10 min at 4 °C. The supernatant was then ultracentrifuged at 120,000 × g for 45 min at 4 °C. The pellet was resuspended in a buffer containing 50 mM Na_3_PO_4_, 300 mM NaCl, and 8% (w/v) sucrose (pH 7.0). The supernatant was placed in a buffer containing 50 mM Na_3_PO_4_, 300 mM NaCl, and 40% (w/v) sucrose (pH 7.0), and was centrifuged at 140,000 × g for 90 min at 4 °C. The band containing the membrane fractions was collected and ultracentrifuged at 180,000 × g for 30 min at 4 °C. The resultant pellet was resuspended in 160 mM of NaCl. The amount of total protein within the crude cell membrane fraction was estimated using the bicinchoninic acid (BCA) method.

Crude cardiac SRs were obtained from canine heart homogenate via standard techniques of differential centrifugation^45^. The resultant pellet was resuspended in a solution containing 600 mM KCl, 300 mM sucrose, 30 mM imidazole, and 1 mM DTT (pH 7.2) to yield a final protein concentration of 5–20 mg/ml.

The crude membrane fractions containing the target ion channels were filtered using an extruder kit with a pore diameter of 0.2 μm (Avanti Polar Lipid, Alabama, USA) to obtain a vesicle homogeneous in size and increase fusion efficiency using the BLM.

### Western blot analysis of the crude membrane faction containing the target ion channels

One volume of sample buffer (Laemmli Sample Buffer, BIO-RAD, California, USA) was added to samples of the crude membrane fraction. The samples containing the target ion channels were separated by SDS-PAGE using 7.5% SDS-PAGE gel and transferred to PVDF membranes. The PVDF membranes were treated with 0.5% (w/v) skim milk in a TBS-T (20 mM Tris, 500 mM NaCl, pH7.5, 0.1% (v/v) Tween 20) buffer for blocking. The membranes were reacted with a primary antibody in TBST. The following antibodies were used: anti-hERG rabbit polyclonal IgG. After washing the primary antibody, the membranes linked with primary antibody were bound with horseradish peroxidase conjugated mouse anti-rabbit IgG. The horseradish peroxidase was reacted by ECL Prime Western Blotting Detection Reagents (GE Healthcare, Illinois, USA) and the band of target ion channels was detected using ChemDoc XRS + system (BIO-RAD).

### Ion channel current measurement on crude cell membrane fractions

The BLM was formed using the droplet contact method^10,11^. Briefly, lipid dissolved in *n*-decane (20 mg/mL DOPC/DOPE = 3:1 molar ratio, 3.8–5.5 μL) was added to each double well on the chip. A buffer solution, 20–23 μL, or a buffer solution containing diluted crude cell membrane fractions, 20–23 μL, was then added to each well. The BLM was spontaneously formed at the microapertures on the separator integrated into the double well. The ion channels were reconstituted in the BLM by fusing with the membrane fraction. The buffer solutions in the double well were electrically connected with the recording and the ground electrodes via Ag/AgCl, and were called droplet A and droplet B, respectively. We chose buffer compositions similar to the physiological conditions of each ion channel. For example, the composition of the buffer for the hERG channels was 10 mM HEPES and 120 mM KCl (pH 7.2) for both droplets A and B. The detailed conditions for each ion channel used in this work are shown in Table 1.

The current signals of the ion channel were recorded using a multichannel patch clamp amplifier with a 1-kHz low-pass filter at a sampling frequency of 5 kHz (Tecella JET, California, USA). The measurement temperature was 23 ± 1 °C. Current analysis was performed using the pCLAMP software program (Molecular Devices, California, USA).

## Electronic supplementary material

Supplementary Information
