# Supplementary material for: Electrophysiological measurement of ion channels on plasma/organelle membranes using an on-chip lipid bilayer system
Source: Sci Rep. 2018 Nov 30;8:17498. doi: 10.1038/s41598-018-35316-4 (PMC6269590; doi:10.1038/s41598-018-35316-4)
Supplement: Supplementary file 1 — Supplementary Information [file 41598_2018_35316_MOESM1_ESM.docx]

**Electronic Supplementary Information**

Electrophysiological measurement of ion channels on plasma/organelle membranes using an on-chip lipid bilayer system

**Koki Kamiya^1^, Toshihisa Osaki^1,2^, Kenji Nakao^3^, Ryuji Kawano^1^, Satoshi Fujii^1^, Nobuo Misawa^1^, Masatoshi Hayakawa^4^, Shoji Takeuchi^1,2*^**

^1^Artificial Cell Membrane Systems Group, Kanagawa Institute of Industrial Science and Technology, 3-2-1 Sakado Takatsu-ku, Kawasaki, Kanagawa 213-0012, Japan.

^2^Institute of Industrial Science, The University of Tokyo, 4-6-1 Komaba, Meguro-ku, Tokyo 153-8505, Japan.

^3^ Biomolecular Research Laboratories, Pharmaceutical Research Division, Takeda Pharmaceutical Company Ltd., 2-26-1 Muraokahigashi, Fujisawa, Kanagawa 251-8555, Japan

^4^Research and Development Department, Kanagawa Institute of Industrial Science and Technology, 3-2-1 Sakado, Takatsu-ku, Kanagawa 213-0012, Japan

*Corresponding author:

Shoji Takeuchi

Artificial Cell Membrane Systems Group, Kanagawa Academy of Science and Technology, KSP East 303, 3-2-1 Sakado, Takatsu, Kawasaki, Kanagawa 213-0012, Japan, and Institute of Industrial Science, The University of Tokyo, 4-6-1 Komaba, Meguro-ku, Tokyo 153-8505, Japan

Phone: +81-3-5452-6650

Fax: +81-3-5452-6649

Email: takeuchi@iis.u-tokyo.ac.jp

**
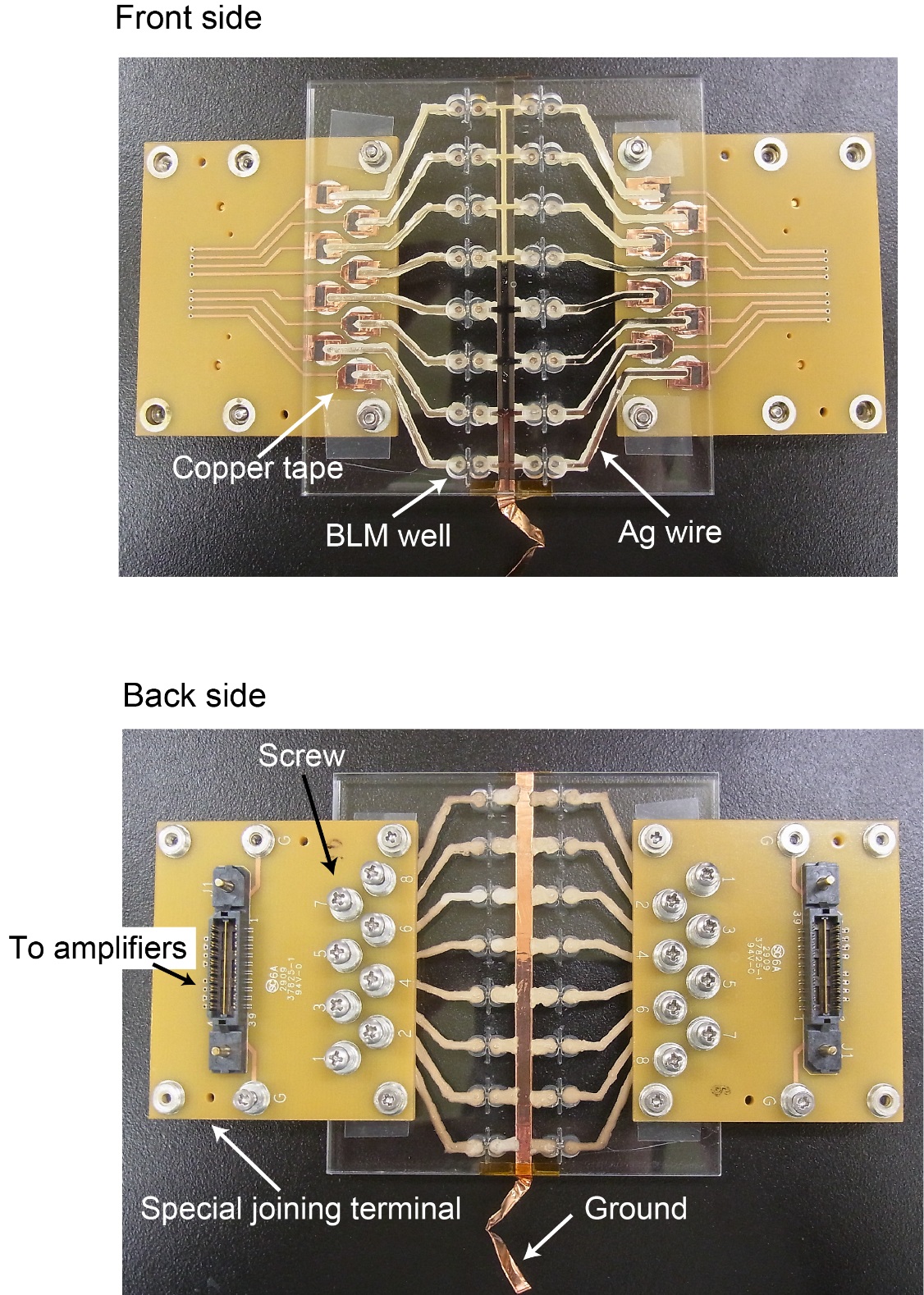
**

**Figure S1.** Previous 16-ch BLM chip with the Ag wires and special joining terminals.

**
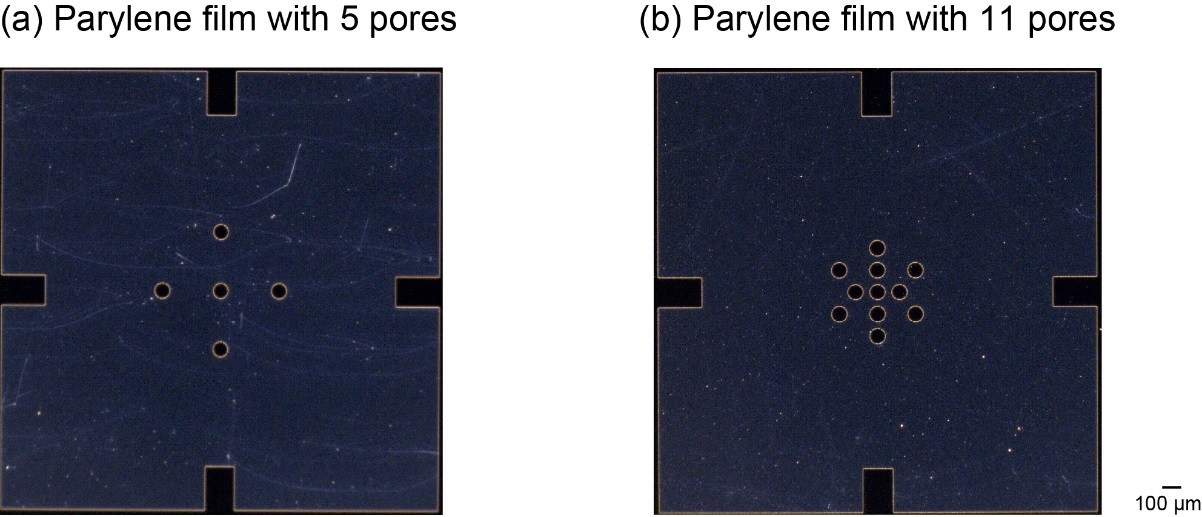
**

**Figure S2.** Parylene film with 5 pores (a) and 11 pores (b).

**
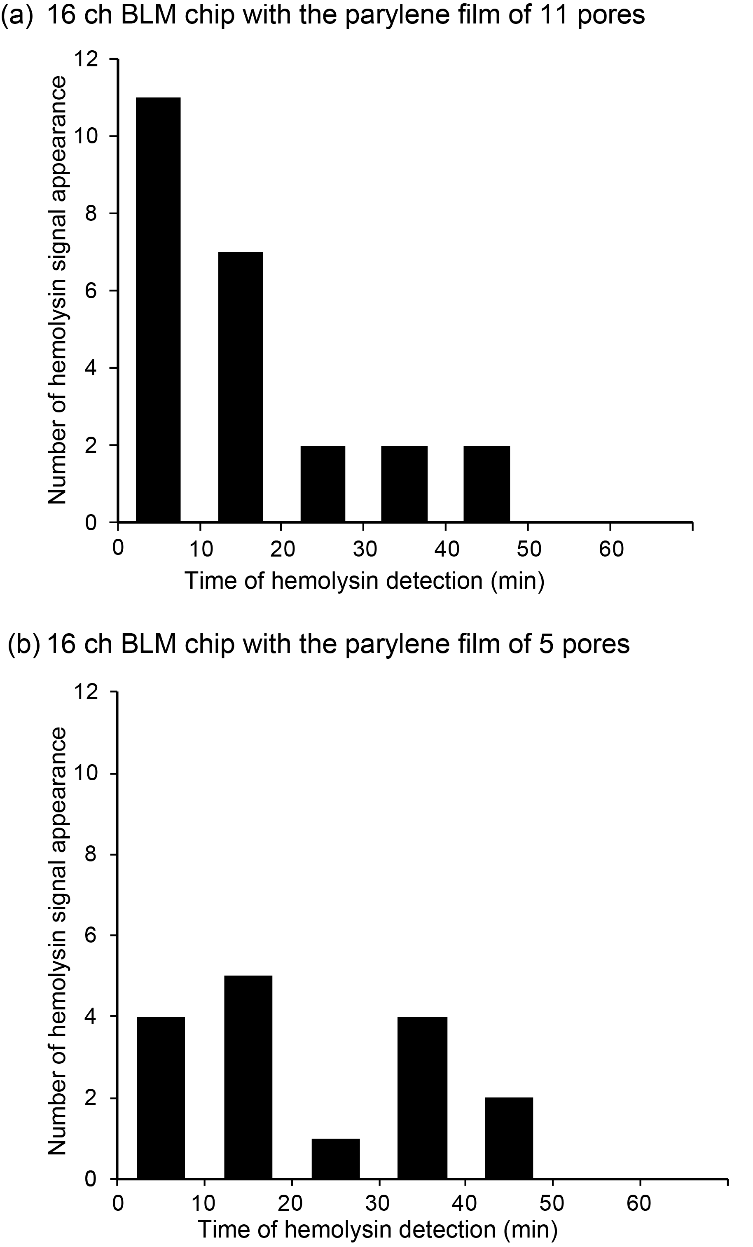
**

**Figure S3.** Time of the initial α-hemolysin current signal detection using two different 16ch BLM chips with either 11 pores and 5 pores (N=3). We measured α-hemolysin signals using each BLM chip at three experiments. The number of signal detection into 48 wells were 24 wells (11 pores) and 16 wells (5 pores). The average time of initial signal detection were 16.5 ± 14.1 (s.d.) min (11 pores) and 21.5 ± 13.6 (s.d.) min (5 pores).


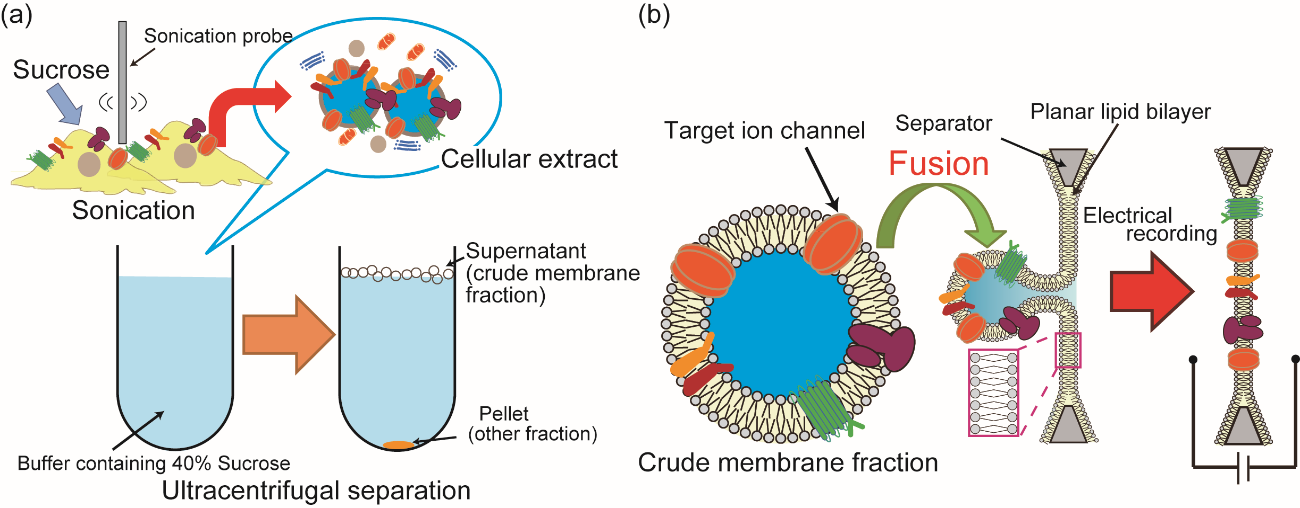


**Figure S4.** (a) Schematic of the method used to isolate the crude cell membrane fractions containing the target ion channels. Cells that overexpress ion channels were prepared and the crude cell membrane fractions were obtained using sonication and centrifugation. (b) The crude cell membrane fractions were fused with the planar lipid bilayer formed by the droplet contact method. Thereby, ion channels were reconstituted to the planar lipid bilayer.

**
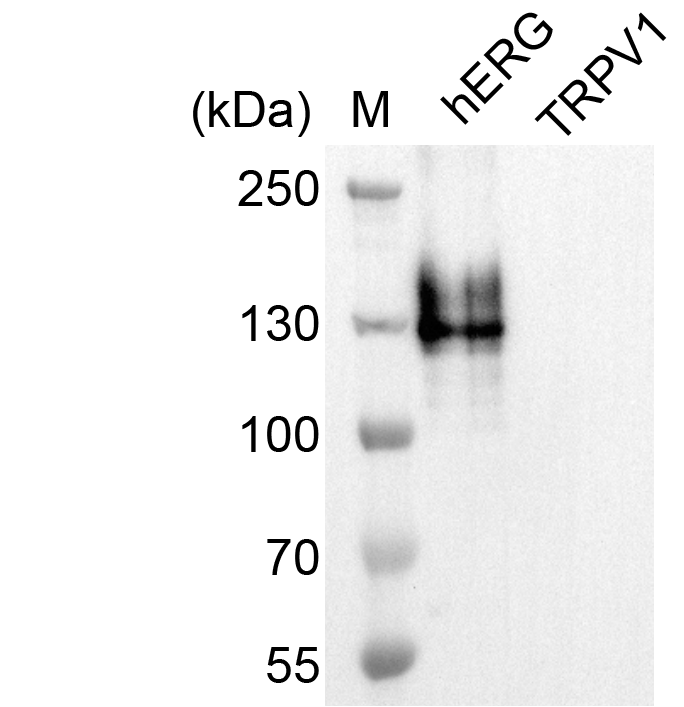
**

**Figure S5.** Western blot analysis of the hERG channels. M: marker. hERG: the crude membrane fraction containing hERG. TRPV1: the crude membrane fraction containing TRPV1.


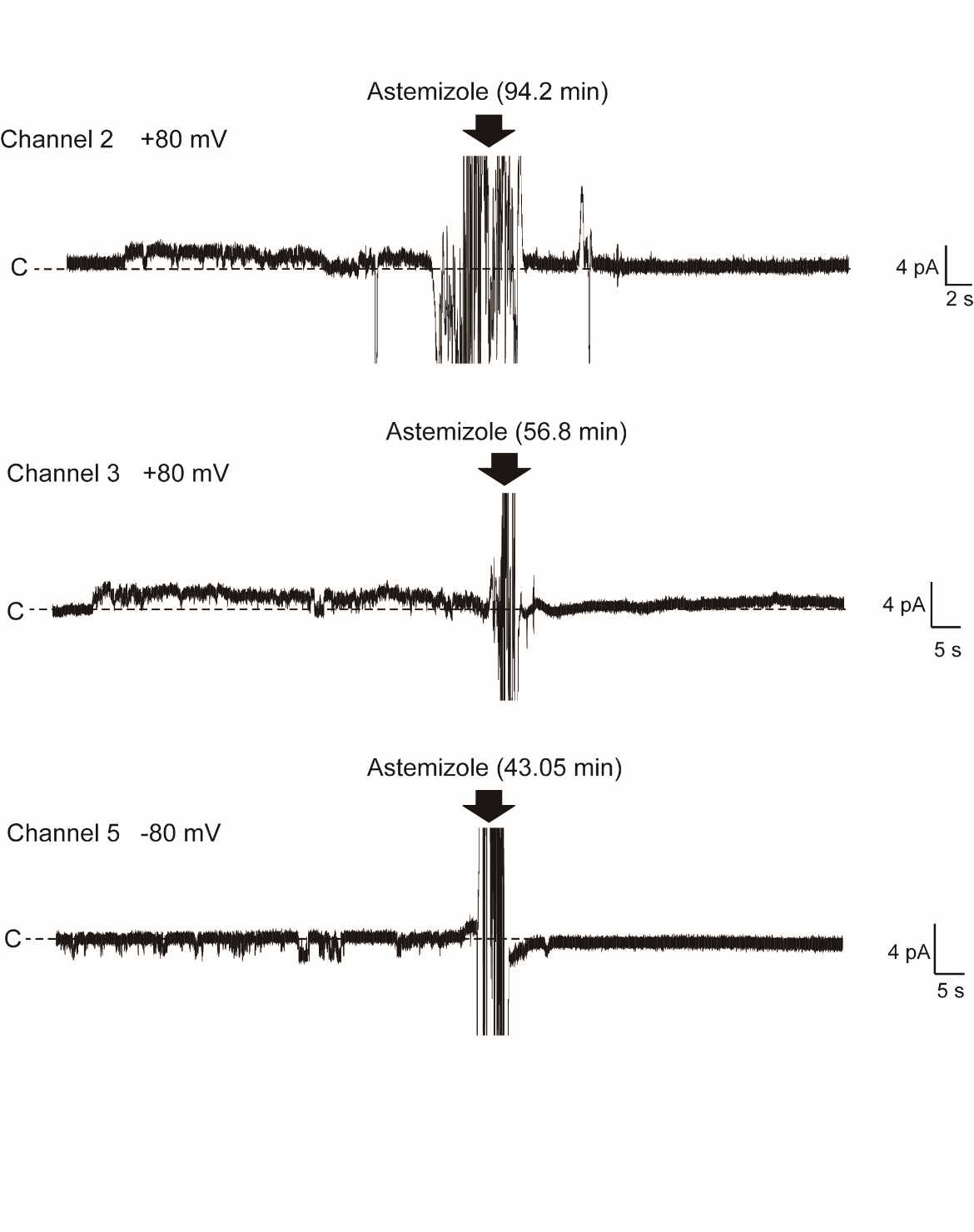


**Figure S6.** Inhibition assay of hERG channel activity using our 16-ch BLM on-chip array. The final concentration of 100 nM astemizole was added to the well at different times and voltages.

**
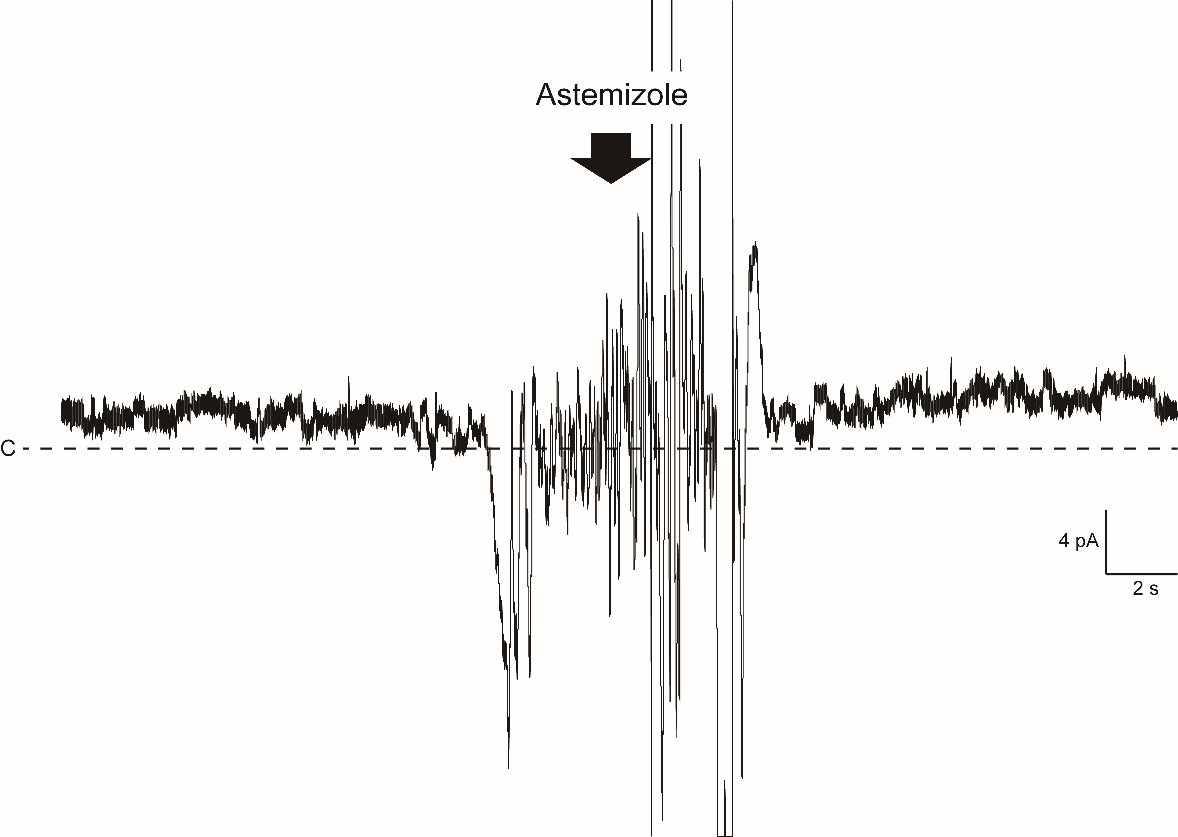
**

**Figure S7.** Current traces of the crude cell membrane fraction from free style 293 cells without hERG channels. The final concentration of 100 nM astemizole was added to the well. Applied potential: +80 mV.


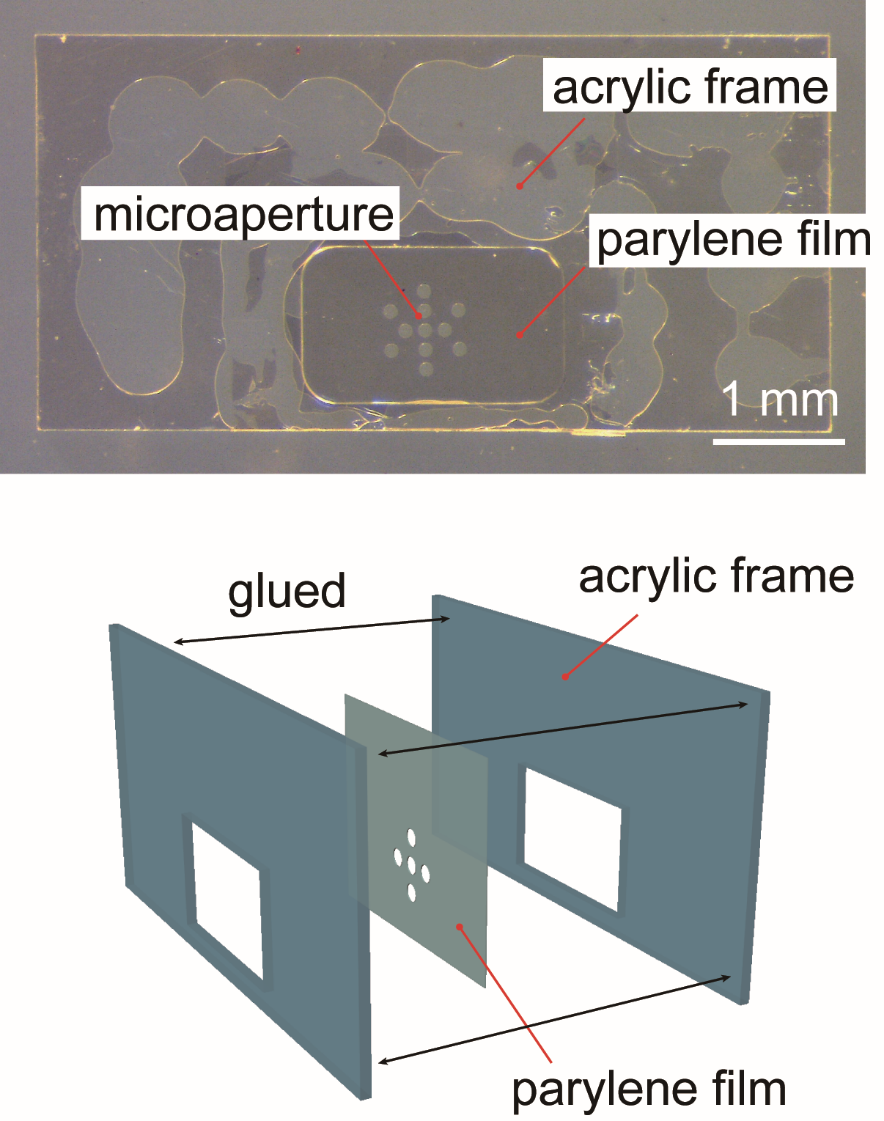


**Figure S8.** Micrograph of the separator. Thin parylene film was supported by the acrylic frames (bottom).
